# Explaining neural activity in human listeners with deep learning via natural language processing of narrative text

**DOI:** 10.1038/s41598-022-21782-4

**Published:** 2022-10-25

**Authors:** Andrea G. Russo, Assunta Ciarlo, Sara Ponticorvo, Francesco Di Salle, Gioacchino Tedeschi, Fabrizio Esposito

**Affiliations:** 1 Department of Advanced Medical and Surgical Sciences, School of Medicine and Surgery, University of Campania “Luigi Vanvitelli”, Piazza Luigi Miraglia, 2, 80138 Naples, Italy; 2 Department of Medicine, Surgery and Dentistry, “Scuola Medica Salernitana”, University of Salerno, Baronissi, Salerno, Italy; 3 Center for Magnetic Resonance Research, Department of Radiology, University of Minnesota, Minneapolis, MN USA; 4 Department of Diagnostic Imaging, University Hospital “San Giovanni di Dio e Ruggi D’Aragona”, Salerno, Italy

**Keywords:** Functional magnetic resonance imaging, Language

## Abstract

Deep learning (DL) approaches may also inform the analysis of human brain activity. Here, a state-of-art DL tool for natural language processing, the Generative Pre-trained Transformer version 2 (GPT-2), is shown to generate meaningful neural encodings in functional MRI during narrative listening. Linguistic features of word unpredictability (surprisal) and contextual importance (saliency) were derived from the GPT-2 applied to the text of a 12-min narrative. Segments of variable duration (from 15 to 90 s) defined the context for the next word, resulting in different sets of neural predictors for functional MRI signals recorded in 27 healthy listeners of the narrative. GPT-2 surprisal, estimating word prediction errors from the artificial network, significantly explained the neural data in superior and middle temporal gyri (bilaterally), in anterior and posterior cingulate cortices, and in the left prefrontal cortex. GPT-2 saliency, weighing the importance of context words, significantly explained the neural data for longer segments in left superior and middle temporal gyri. These results add novel support to the use of DL tools in the search for neural encodings in functional MRI. A DL language model like the GPT-2 may feature useful data about neural processes subserving language comprehension in humans, including next-word context-related prediction.

## Introduction

Deep artificial neural networks and deep learning (DL) tools can be remarkably accurate in assisting or performing human tasks and can therefore be used to replicate the output of human reasoning in a computer simulation. Such DL “in-silico” models are increasingly used in neuroscience to address some of the complex neural mechanisms that subserve high-order cognitive functions in humans^1,2^. This can be done empirically by applying the same information as input (under the same or a different modality) to both the artificial network and the biological system and then extracting suitable “explanation” parameters from the former to predict the neural responses measured in the latter^3^. In the field of natural language processing (NLP), DL models have been successfully combined with neuroimaging techniques to recognize and localize some specific neural mechanisms putatively subserving language processing in the human brain^4–7^.

Among the available NLP models based on DL, Schrimpf et al.^7^ showed that the Generative Pre-trained Transformer version 2 (GPT-2)^8^ has the best performance in the encoding of neural signals. The architecture of the GPT-2 is based on the so-called “attention mechanism”^9^ and, compared to other highly performative attention-based DL models, such as BERT^10^, it uses only the information on the left side to predict the next word in the text^8^. Recently, Goldstein et al.^5^ used electrocorticography (ECoG) to demonstrate that the GPT-2 shares similar computational principles with the human brain when processing text.

The prediction of the next word from a given sequence also occurs in the human brain when continuously engaged in the generation of meaningful linguistic structures from the auditory streams of words perceived up to that moment, such as during natural listening^6,11,12^. In addition, it is an established principle of sensory perception that an attention mechanism is also needed in the human brain to improve the prediction mechanism via synergistic modulation of input signals^13,14^. Thus, if a similar principle applies to sequences of tokens (i.e., words, punctuations, etc.), feeding the GPT-2 with a given text, and analyzing its internal process, might help to explain some of the neural processes elicited in the brain of human listeners to whom the same text is narrated.

So far, previous studies have mostly focused on abstracting the neural representation of specific linguistic content from the embeddings of the GPT-2 model or on obtaining the empirical estimation of next-word probabilities from the output of the model^4–6^. Moreover, one preliminary study by Kumar et al.^15^ showed the possibility of explaining the cascade of cortical computations during language comprehension by giving in input a text to the BERT model^10^ and leveraging the output of its so-called attention heads, which are the essential internal components of an attention-based DL model, that operate directly on the input words^15^. However, no previous studies considered the model “reasoning”, i.e., how the model evaluates the input words and assigns the responsibility to each of them for generating a given output^16^. To address this problem, the “input saliency” methods^16^ can be used to generate the so-called saliency score, which is a simple weight assigned to each word of the input sequence reflecting how much that word was important for the model in the prediction of the next word^16,17^.

The aim of this work is to evaluate the possibility of using metrics derived from a DL model, including ones that aim at explaining its detailed internal reasoning, to provide additional insights on the neural mechanisms underpinning language comprehension in humans. We show that the saliency scores, as obtained from feeding a pre-trained GPT-2 with the transcription of a 12-min spoken narrative, significantly explained the neural signals, as measured with functional MRI (fMRI), in the brain of human listeners who listened to it. To this purpose, a previously published naturalistic fMRI data set^11^, from a group of Italian participants listening to a story in both forward (FW) and backward (BW) conditions, was re-analyzed using two GTP-2 derived metrics: the negative logarithm of the next-word probability (i.e., the surprisal^18^) and the saliency scores associated with all input words in segments of text (i.e., the context words). These measures were calculated by feeding the GPT-2 model with input contexts of varying duration, from a minimum of 15 s to a maximum of 90 s of text. The analyses revealed that the GPT-2 surprisal significantly explained the fMRI signals from an extended network of language-related areas across all time windows, whereas the saliency scores were highly selective with respect to the length of context and significantly explained the neural data only for longer time windows, especially in the superior temporal cortex. Thereby, the GPT-2 appears capable of robustly explaining brain activations associated with context-related word prediction, highlighting a mechanism that is likely pivotal to language comprehension in humans.

## Materials and methods

### Participants

The raw experimental data, processed and analyzed in this work, have been acquired in a previous fMRI naturalistic experiment on the neural correlates of the linguistic prediction during spoken narrative listening. Full details can be found in the original paper^11^.

All the volunteers enrolled in the experiment (21 females, mean age 24.5 ± 4.5 years old) were Italian native speakers without known psychiatric or neurological problems, with normal or corrected-to-normal vision and without hearing, developmental and language-related problems. All participants self-reported to be right-handed and were naive with respect to the purpose of the experiment. The study was approved by the Ethics Committee of the University of Salerno and performed in accordance with the Declaration of Helsinki. Each participant signed a written informed consent to participate in the study. No part of the study procedures was pre-registered prior to the research being conducted.

### Stimuli and experimental procedure

Participants listened to a short story in both original and its reversed version, while in the MRI scanner. Reversed audio waveform was selected as control condition as it omits meaning and linguistic components, but it is comparable to forward speech in terms of auditory characteristics^11,12^. To avoid and reduce possible biases due to previous knowledge of the story, in this study a short narrative of an amateur writer was chosen as our stimulus. Indeed, all subjects declared that they did not have any prior knowledge of the story.

Technical details of the stimuli and of the experimental procedure are reported in the Supplementary Materials.

### Image acquisition and functional MRI pre-processing

MRI acquisition was performed with a 3 T scanner (Magnetom Skyra, Siemens Healthcare, Germany). Full details on the sequence parameters can be found in^11^.

MRI data were pre-processed using FLS (https://fsl.fmrib.ox.ac.uk/fsl/fslwiki) and the Data Processing Assistant for Resting-State fMRI toolkit (DPARSF 5.0 http://www.rfmri.org) that is implemented in MATLAB (The MathWorks, Inc., Natick, MA www.mathworks.com) and based on SPM12 (Wellcome Department of Imaging Neuroscience, London, UK, http://www.fil.ion.ucl.ac.uk/spm/). More details are reported in the Supplementary Materials.

### Estimation of the surprisal and saliency scores

In this work a version of the GPT-2 model for the Italian language, called GePpeTto^19^, was used. Although GePpeTto includes 12 layers, thereby corresponding to the smallest version of the GPT-2, at the best of our knowledge it is the only freely available GPT-2 model for the Italian language that has been trained from scratch on a large corpus encompassing different sources and different styles thus ensuring a training on a mix of both standard and less-standard Italian. The model was used without additional fine tuning. More details about the models can be found in Supplementary Materials, whereas a complete description about the model training and testing, the model parameters and its performances can be found in^19^.

The narrative text was first tokenized (i.e., the narrative was split into smaller units, such as individual words or portion of words. See Supplementary Materials for more details) and then these tokens were used to estimate the word-surprisal and corresponding saliency scores. In particular, moving one token at time the whole set of tokens was sampled using a sliding-window approach, with windows spanning from a minimum of 15 s to a maximum of 90 s with a step of 15 s. An interval of 15 s was selected as windows size and step as this reflects the period during which a hemodynamic event evolves^20^. Moreover, this specific size allowed us to provide in input to the GPT-2 model enough information to produce a quite reliable output. In fact, a very recent study^5^ reported a correlation value around 0.75 between the GPT-2’s predictions and human predictions when using the average number of tokens contained in 15 s (see Table 1). Finally, an incremental step of 15 s allowed us to have a range of six time windows that included not only short-term linguistic phenomena but also linguistic phenomena unfolding across sentences and paragraphs.

**Table 1:** Descriptive values of each input window.

Window size (s)	Number of tokens (mean ± s.d.)	Number of words (mean ± s.d.)	Surprisal (mean ± s.d.)	Number of fMRI time points
15 s	53.52 ± 7.2	45.4 ± 5.46	3.76 ± 1.64	683
30 s	105.62 ± 15.36	89.55 ± 12.02	3.69 ± 1.171	668
45 s	156.47 ± 26.24	132.56 ± 20.94	3.8 ± 1.87	653
60 s	206.1 ± 39.17	174.61 ± 31.42	3.87 ± 1.93	638
75 s	254.51 ± 53.64	215.53 ± 43.17	3.93 ± 1.97	623
90 s	301.59 ± 69.23	255.32 ± 69.23	3.99 ± 2.02	608

A set of context tokens (i.e., all the previous tokens contained in the time window) were assigned to each available token (n = 2512) and provided in input to the GPT-2 model to estimate both the surprisal value and the associated saliency scores. The size of theses input contexts varied across time windows and tokens. Therefore, for each time window, the sizes of all the input contexts, expressed both in number of tokens and number of (meaningful) words, were averaged across all the available tokens and reported in the second and third column of the table.

Raw surprisal values estimated for each token, were first convolved with the hemodynamic response, and then down sampled to the number of available fMRI time point (i.e., 709). The average surprisal value across the fMRI time points are reported in the fourth column of the table.

The use of a sliding window approach and the variable size of the time windows allowed the use of a reduced subset (i.e., lower than 709) of fMRI time points for the saliency score analysis. In the fifth column of the table the number of fMRI time points available for the saliency score analysis is reported for each time window.

Both the surprisal and the saliency scores of each token were estimated by providing in input to the GPT-2 all the previous tokens (i.e., context tokens) contained in that specific fixed time interval.

The notion of surprisal is based on the assumption that the language comprehension system, after the processing of the first t−1 items (i.e., in our case, the context tokens) will be in a state that implicitly assigns a conditional probability to each potentially upcoming item^21^, thereby the surprisal of a token is defined as:

1 $${\text{surprisal}}\left( {{\text{token}}} \right) = - \;{\text{log1}}0({\text{P}}\left( {{\text{token}}|{\text{context tokens}}} \right)$$

If the conditional probability of the observed item is one, it means that given its left-side context there are no other possible items than the actual one, thereby the surprise in observing it is null. Previous studies have shown that the surprisal is parametrically linked to the language-related cognitive effort or the linguistic processing difficulty^22,23^ and it can be successfully used to predict neural responses during narrative listening^11,12^.

In this study, the conditional probability in formula (1) was estimated by (i) providing the context tokens in input to the GPT-2, (ii) extracting and normalizing the tensor associated with the last context token and (iii) selecting the probability associated with the actual next token in the narrative. In fact, the extracted tensor contained the probability of occurrence at that point of each token in the model vocabulary. Because of the sliding window approach, the surprisal value of the first N tokens, where N is the length of the time window, was estimated by considering only the available previous tokens.

This procedure was repeated for each token and for all the six time windows.

A similar procedure was used to estimate the saliency scores. However, while the surprisal estimation provided a single value for each token, the estimation of the saliency scores returned a vector of N values reflecting the importance (the “weight”) of each context token for the prediction of the current token, thus, aiming to fulfil the need of explaining the model output^16^. While in the NLP field the term “saliency” is quite commonly used to describe the marginal effect of each input token on the prediction^16^, in the linguistic field it is used to indicate a diverse range of phenomen^24^. In this study, we referred to the interpretation more aligned (and similar) to the description from the NLP field, i.e., that a salient token exerts an influence on the next-token prediction by making certain upcoming input more expected^24^ (for a more detailed review on the associations among surprisal, attention and salience in the language processing please see^24^).

The saliency scores vector for each token, indicating the importance of each context token in the prediction of the current token, was obtained with the “GradientXInput” method^25^, using the following formula:

2 $${\text{Saliency vector}} = \left| {\left| {{\text{Grad}}_{{{\text{Xi}}}} \left( {{\text{f}}_{{\text{c}}} \left( {{\text{X}}_{{{1}:{\text{n}}}} } \right)} \right){\text{X}}_{{\text{i}}} } \right|} \right|_{{2}}$$

where X_i_ is the embedding vector of the current/predicted token at position i, and Grad_Xi_f_c_(X_1:n_) is the backpropagated gradient of the score assigned to the predicted token^25^.

To obtain a single value for each context token, we used the L2-norm as it has been observed that the “GradientXInput” method in combination with the L2-norm performs better than other methods in tasks related to output explainability^26^. Finally, the saliency scores values were normalized by dividing them by their sum^27^.

This procedure was repeated for each token and for all the six time windows.

### Functional MRI data analysis

Both surprisal values and saliency scores vectors were used as predictors of interest in two separate GLM where the dependent variable was the average neural signal of the 1000 region-of-interest (ROI) of the 7-networks Schaefer's parcellation^28^ for both speech conditions. Beside the predictor of interest, other relevant linguistic features such as word duration (WD), lexical frequency (LF), and word sound (WS) were considered. In fact, previous studies reported significantly positive activations for the LF (when contrasting speech conditions)^11^ and for the WS (for both speech conditions)^29,30^ in naturalistic fMRI experiments.

An ROI-based analysis was selected (over a voxel-based analysis) to mitigate the computational costs (especially in the case of saliency scores analysis). For both GLM analyses only the time points characterized by the presence of the audio stimulus were retained, thus resulting in a set of functional data of 709 volumes, and the contrast between the FW and the BW conditions was evaluated.

All the statistical analyses were repeated six times by using each time the surprisal values and the saliency scores associated with a specific time window. Resulting statistical results were considered significant at *p* < 0.05 corrected with the Bonferroni criterion (considering n = 1000 comparisons), then were stored in a volumetric map (where they were assigned to the corresponding atlas’ parcellation) that was eventually projected onto an inflated brain surface in the MNI space for visualization purposes using CAT12 (https://neuro-jena.github.io/cat/).

More details on both the workflow to create suitable fMRI predictors starting from the raw values of surprisal and saliency and the statistical analyses are reported in the Supplementary Materials.

**Figure 1:**
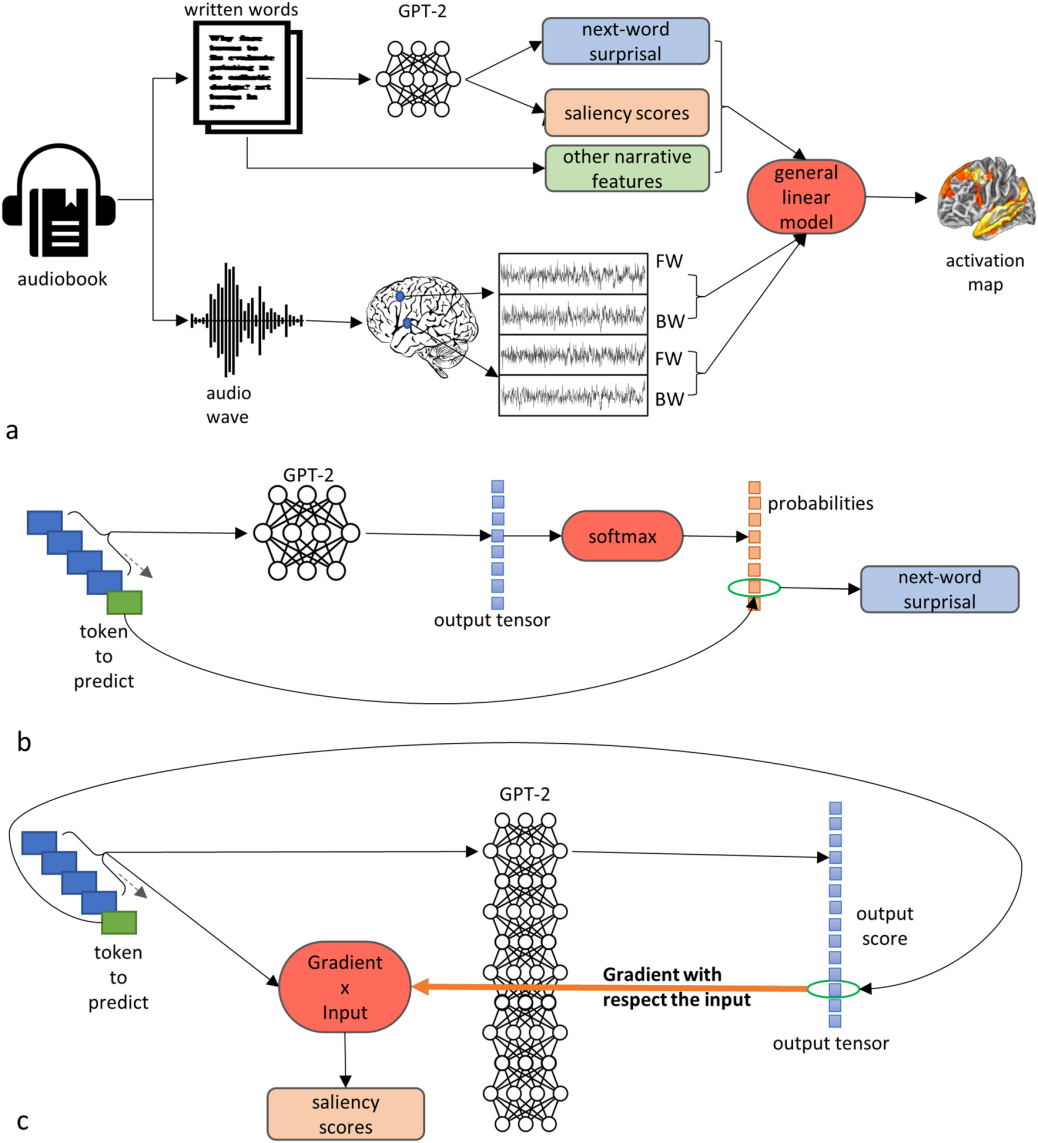
Graphical description of the analysis workflow. The text of the audiobook was first tokenized and then given in input to the GPT-2 model. For each window, the surprisal value associated with the next token in the text and the saliency scores associated with each context token were estimated. The resulting values were first processed to generate suitable predictors and then used to encode the fMRI signal elicited by both listening conditions (FW and BW) in a mixed-effects GLM group analysis (**a**). The conditional probability used in formula (1) to estimate the surprisal was estimated by providing the context tokens in input to the model, extracting and normalizing via softmax the tensor associated with the last context token and, finally selecting from it the values associated with the actual next token in the narrative (**b**). The saliency scores associated to each context token used to predict the next token were estimated using the “GradientXInput” method by multiplying the embedding vector of the token to predict with the back-propagated gradient of the score assigned to it by the model (**c**). Credit for the icons from NounProject.com contained in the figure: “Sound wave” by Rabee Balakrishnan; “Audiobook” by Vectors Market; “Document” by Brian Gonzalez; “Brain” by by Clockwise; “Neural Network” by Chenyu Wang.

A graphical overview of the workflow is presented in Fig. 1.

## Results

### Neural data modeling with the GPT-2

The tokenization procedure resulted in 2512 tokens of which only a subset of 1589 were full words. Both surprisal and saliency scores were estimated for all the six time windows, thus the GPT-2 was fed with a minimum of 53.52 ± 7.2 (mean ± std) tokens for the shortest time window (15 s) and with a maximum of 301.59 ± 69.23 tokens using the largest time window (90 s). The number of tokens feeding the GPT-2 varied not only across time windows (i.e., longer time windows contained more tokens) but also across tokens because of intrinsic narrative factors such word length, speaking frequency, and speaker intonation (Table 1). The variable size of the GPT-2 input influenced the estimation of the surprisal of the next token and a significantly positive correlation was observed between the average number of available tokens for each time window and the corresponding average surprisal (r = 0.93, *p* = 0.007), therefore suggesting a higher uncertainty of the model with larger number of input tokens possibly due to a higher heterogeneity of the input itself.

Finally, the increase of the size of the time window and the sliding window approach also influenced the number of fMRI time points usable for the saliency scores analysis, as with longer time windows more fMRI time points were excluded from the analysis compared to shorter time windows (Table 1).

### Surprisal analysis

Across the time windows, the average surprisal varied from a minimum (mean ± std) of 3.69 ± 1.71, when using the time window of 30 s, to a maximum (mean ± std) of 3.99 ± 2.02 when using the time window of 90 s (Table 1).

Regional activation patterns elicited by the surprisal analysis were spatially consistent across all six time windows, thereby corroborating most previous works that already leveraged the outputs of the GPT-2 model with different input context lengths^4–7^. In total, 144 out of 1000 ROIs were significantly activated (*p* < 0.05 Bonferroni corrected), in at least one time window, and most of them (40 ROIs) were significantly activated in all the time windows (see supplementary Table 1 for more details). The brain patterns associated with different time windows differed in the number of activated ROIs, ranging from a minimum of 46 ROIs (time window of 60 s) to a maximum of 121 ROIs (time window of 15 s) (Supplementary Table 1).

**Figure 2:**
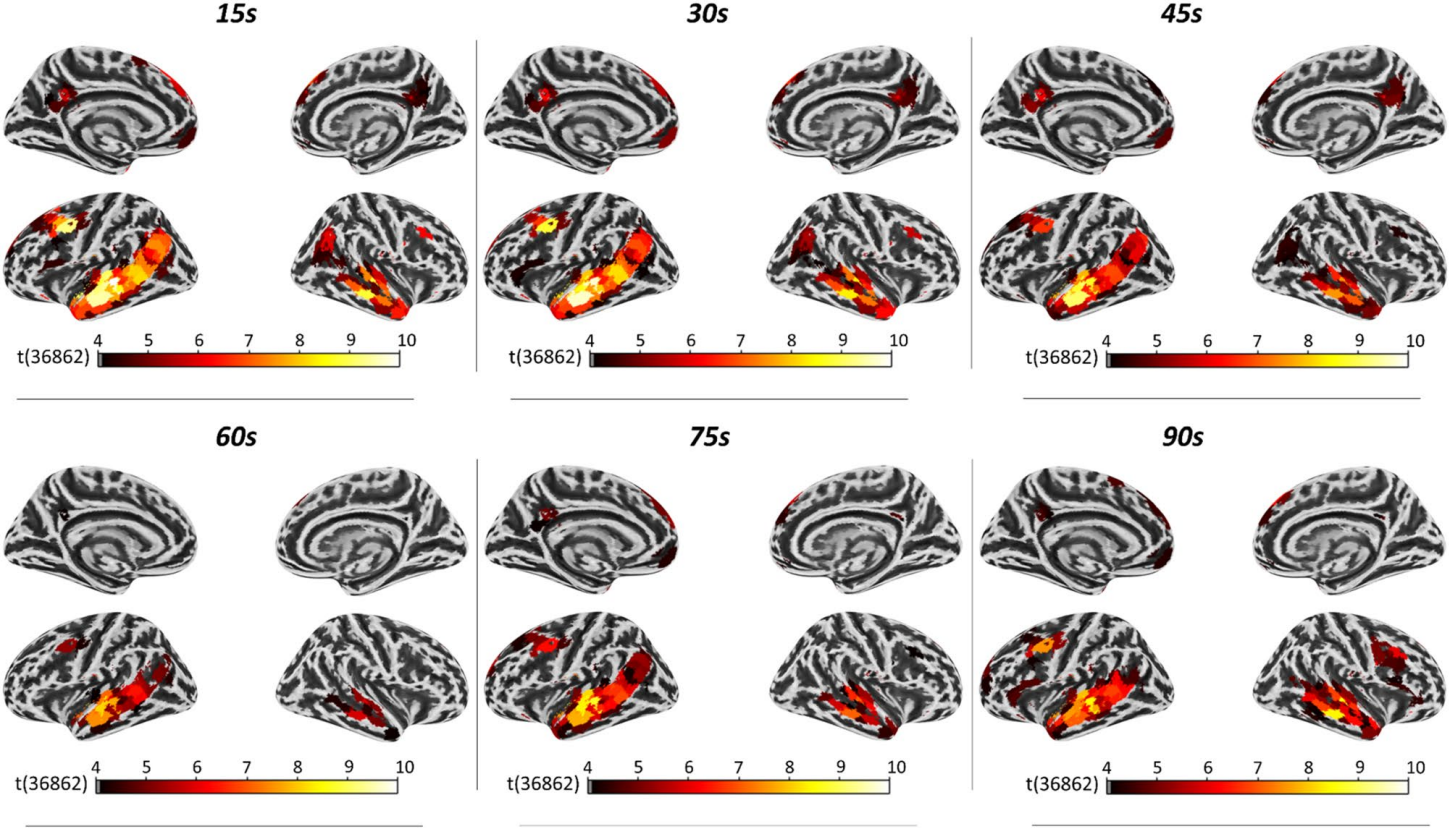
Regional statistical map of the contrast FW versus BW projected on a surface in the standard MNI space (left and right hemisphere). Color-coded areas indicate the atlas’ parcellations that yielded a significantly higher correlation with the surprisal predictor in the FW speech condition compared to the BW speech condition (*p* < 0.05, Bonferroni corrected). A left lateralized regional pattern, encompassing parcellations mainly in the temporal and frontal areas, was observed for all the time windows. Consistently across the time windows, the highest statistical values were observed in parcellations located in the left superior/middle temporal gyrus.

The six brain patterns overlapped in the middle and superior temporal gyri (bilaterally), the anterior and posterior cingulate cortex, and the left prefrontal cortex. In line with previous findings ^4,6,11,12^, the strongest effects for the surprisal were observed in ROIs located in the left superior and middle temporal gyrus (Fig. 2, see supplementary Table 1 for more details), thus confirming the putative role of these ROIs in human language comprehension via the word prediction mechanism^31^. The involvement of higher-order areas in frontal and parietal lobes could be further explained by the use of time windows that spanned many seconds of the text and, therefore, were large enough to include sentences and/or paragraphs^30^.

### Saliency score analysis

**Figure 3:**
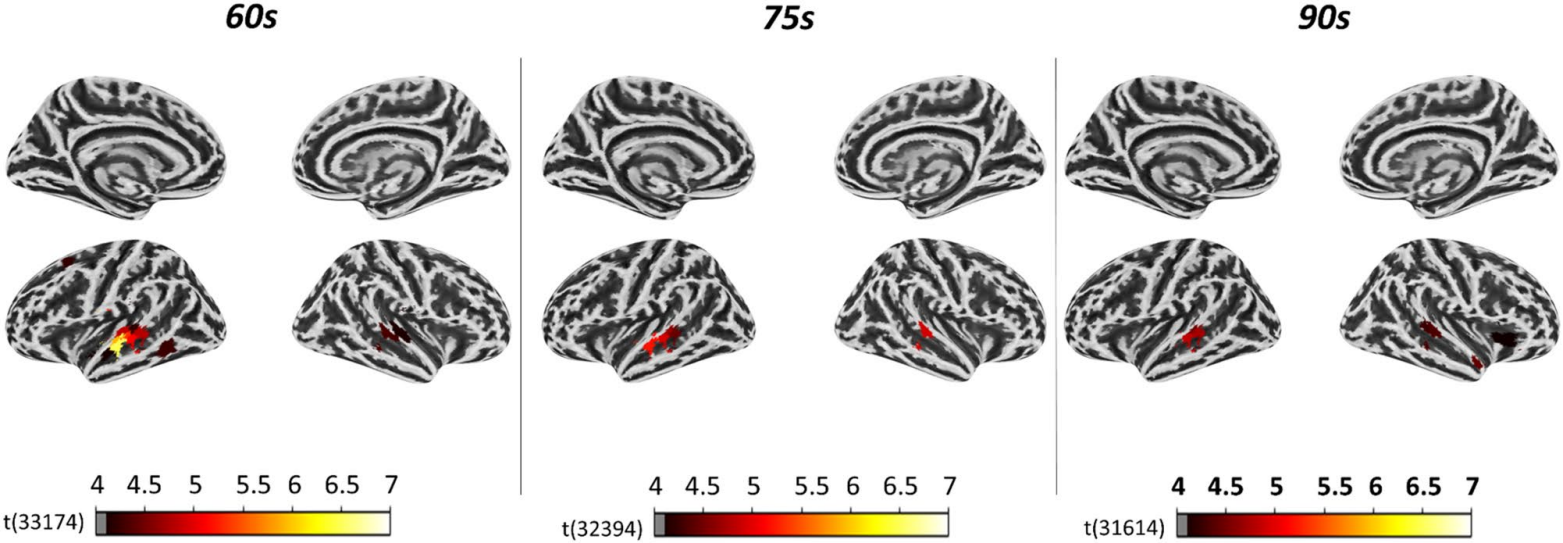
Regional statistical map of the contrast FW versus BW projected on a surface in the standard MNI space (left and right hemisphere). Color-coded areas indicate the atlas’ parcellations that yielded a significantly higher correlation with the saliency scores predictor in the FW speech condition compared to the BW speech condition (*p* < 0.05, Bonferroni corrected). A left lateralized regional pattern, encompassing parcellations mainly in the temporal areas, was observed only for the three largest time windows (i.e., 60, 75, and 90 s), whereas no significant atlas’ parcellation was observed when considering shorter time window (i.e., 15, 30, and 45 s). Consistently across the time windows, where significant parcellations were observed, the highest statical values were elicited in regions located in the superior/middle temporal gyrus.

Significant effects of the saliency scores were only observed for the three longest time windows (i.e., 60, 75, and 90 s). In total, 17 ROIs out of 1000 (*p* < 0.05, Bonferroni corrected) were found significantly activated in at least one of the three longest time windows. Most ROIs were activated for the 60 s time window but only 12 ROIs were activated for one specific time window (see Supplementary Table 2 for all details). Across the time windows, the strongest effects (i.e., highest statistical values) were observed in ROIs located in the left temporal superior and middle temporal gyri (Fig. 3, Supplementary Table 2).

These findings indicate that the saliency scores derived from the GPT-2 model selectively capture specific neuromodulatory processes occurring in the temporal cortex of the listeners. Considering the role of these scores in the artificial network, the salience-related effects would signal the activation of a similar weighing mechanism in both the artificial network and the brain. To the extent this mechanism provides a window into how the artificial network accounted for the relevance of previous context words, our findings suggest that the sensory prediction model internal to the brain is similarly updated: in practice, this mechanism allows accounting for the different share of responsibility of previously stored words when collecting new sensory evidence for the generation of a prediction error^14^. Thereby, while the magnitude of the prediction error is indexed by the surprisal, the weighting of previous words is indexed by the salience scores. A similar process had been already postulated as necessary in word prediction to enable text comprehension in humans^24^ and, according to our data, it would be putatively hosted in the superior and middle temporal areas. The fact that also the surprisal showed the strongest effects in these areas further supports the idea that context-related word evaluation promotes the neural encoding of word prediction where (and probably how) this takes place.

In contrast to the surprisal analysis, the neural patterns associated with the saliency scores were not consistent across the six time windows.

## Discussion

Recent studies have shown that DL language models based on the transformer architecture^9^, such as the GPT-2, have remarkable performances in explaining the neural correlates of language comprehension and likely share some computational principles with the human brain^4–6,15^. In this work, we provided further support to this hypothesis, and contributed some novel insights about two basic neural mechanisms underpinning language comprehension in humans: word prediction and context-related language processing.

The contribution of this research is two-fold: first our results illustrate with a novel methodology how it is possible to expose an attention-based neural network and a group of human listeners to the same narrative and simulate a prediction mechanism from the former to generate a brain activation pattern from the latter. In particular, our results corroborate the idea that word-level prediction is accurately indexed by the surprisal metric^4,6,11,12^, even if this metric is conveniently estimated from empirical simulations with an NLP model, such as the GPT-2, rather than using a stochastic language model. Second, we showed that it is possible to explore the internal reasoning of the “black box” of a DL language model and derive corresponding metrics to map the neural mechanism underlying language comprehension. In fact, while the saliency scores represent a useful tool for the developers to understand the behavior of a DL model, obtaining a more “explainable” artificial intelligence (AI) tool^16,27^, our findings demonstrate that these scores could be also used as neural features to shed light on neural mechanisms that are either partially unknown or simply not yet (or not completely) modeled by other metrics^15^. Furthermore, our findings are in line with a recent study demonstrating (with a different neuroimaging technique) that (i) the neural responses before the onset of a word contain valuable information about the incoming word, (ii) such information can be suitably extracted from the GPT-2 using its contextual embeddings^5^. In addition, the observed neural activation from the saliency scores clearly suggests the co-occurrence of a weighing mechanism operating on the context words, previously hypothesized as necessary to language comprehension^24^, and here shown as highly similar to a mechanism of input evaluation implemented in a highly performative NLP tool. Moreover, the involvement of areas in the middle and the superior temporal gyrus agrees with previous studies supporting that core aspects of language comprehension, such as keeping intermediate representations active in working memory and predicting upcoming words, do not necessarily engage areas in the executive control (also called “multiple-demand”^32^) network but are instead carried out by language-selective brain areas^33,34^ that, in our case, are the ones relatively earlier in the processing hierarchy. The lack of significant ROIs for the three shortest time windows (i.e., 15, 30, and 45 s) suggests that this weighing mechanism is manifest only when it would be most effective, i.e., over longer time windows when there is a real advantage in accessing an internal model of an evolving discourse^24,35^. Thus, when considering a shorter time window, the observable mechanisms the brain uses to predict the incoming word are not indexed by the varying saliency scores.

In general, considering that the architecture of artificial neural networks was originally inspired by the same principles of biological neural networks, it might be not at all surprising that some specific dynamics observed in the former are somehow reflected in the functioning of the latter, albeit to a different physical scale and/or via different modalities of information exchange (e.g., reading vs. listening). Nonetheless, it is not possible to absolutely warrant this kind of evidence in every circumstance, especially when the depth and complexity of the artificial network increases to levels similar to that of the GPT-2 considered here (or more), and no one-to-one correspondences of computational units is assumed between the model and the brain^2,36,37^. The very same architecture of DL models based on multi-head self-attention modules was not completely inspired by (and does not clearly map to) neural computations in biological networks^38^. Thus, we cannot interpret the computational model as a general cognitive model nor we can reversely infer any critical human features, such as, e.g., abstraction and generalization, for the model ^2^. Nevertheless, it is equally important to not dismiss the potentially useful exchange of information between neuroscience and AI tools as emerging from the analysis of neuroimaging data, especially under naturalistic conditions where even the most detailed a priori cognitive model might be difficult to apply in the explanation of neural responses^1,37^.

Our results will likely inspire new lines of research and applications both in the neuroscientific and AI fields. In general, the idea of using a metric, such as the saliency score, that “explains” the reasoning of the DL model to map the brain functioning could be applied in the process of improving AI models, e.g., to make the human-AI interaction more useful and effective or to lead the model to take more on human-like approach^39^. On the other hand, by inverting the paradigm, another possible application would be the extraction of the stimulus salient features from the brain signal^40^. Finally, the possibility to generate informative predictors using parameters derived from AI models could have relevant clinical applications. For example, the presented methodology could be applied to investigate language dysfunctions in psychiatric and neurodevelopmental disorders, such as schizophrenia^41^ or autism spectrum disorder (ASD)^42^.

Our work comes with some limitations. First, although the GPT-2 model is one of the most biologically plausible models^7^, it has been suggested that it does not reflect the way human beings learn and manage language^43^. Thus, DL models with more human-oriented architecture and learning objective could provide a deeper understanding of language comprehension^1,2,15,43^. Second, we used a text stimulus that was a narrative and that was not familiar for all the listeners. Although, we do not expect different results when using a different kind of stimulus (e.g., news article) or different stories, as both surprisal and saliency scores aim at modeling phenomena beyond the style of the stimulus, future research is needed to investigate the consistency of our predictors in mapping neural response across different stories and text stimulus type. On the other hand, it is plausible that listening to an already known stimulus would engage less the prediction mechanism, thus yielding different results. Thereby, future studies are warranted to assess the influence of the familiarity factor and, especially concerning the saliency scores, also their contribution to the understanding of neural computations in comparison with theoretical approaches^44^. Finally, although fMRI has been successfully used in previous studies to map language processing^4,15,30,43^, the use of high temporal resolution neuroimaging techniques, such as MEG and EEG, could provide results at finer details as their more time-resolved signals could be better suited to a word-based metric such as the salience scores^5,6^.

## Conclusions

In this work, the neural correlates of narrative comprehension in a naturalistic fMRI experiment have been mapped with two metrics estimated with a state-of-art DL language model (GPT-2): the surprisal and the saliency scores. The results observed in the surprisal analysis confirm previous studies and further support the use of DL language models to explain the fMRI signal during spoken narrative listening. The analysis of the saliency scores revealed the presence of a weighing mechanism operating on the listened words which takes place in the superior and middle temporal cortex. As this mechanism explains the performance of an NLP tool in the prediction of new words from the current segment of text, this finding establishes a novel link between the ways the human brain and the chosen DL language model (GPT-2) build up inferences about the next incoming word. This approach works by unpacking the internal reasoning approach of the artificial neural network and, despite the architectural differences, seems to feature a similar mechanism of neural processing in the brain which may inspire novel strategies for addressing human performances in complex cognitive tasks, including natural language comprehension.

## Supplementary Information

Supplementary Information.

## Data Availability

The datasets used and/or analyzed during the current study are available from the corresponding author on reasonable request.
