# Supplementary material for: Explaining neural activity in human listeners with deep learning via natural language processing of narrative text
Source: Sci Rep. 2022 Oct 25;12:17838. doi: 10.1038/s41598-022-21782-4 (PMC9596412; doi:10.1038/s41598-022-21782-4)
Supplement: Supplementary file 1 — Supplementary Information. [file 41598_2022_21782_MOESM1_ESM.docx]

**Supplementary materials**

**Explaining Neural Activity in Human Listeners with Deep Learning via Natural Language Processing of Narrative Text**

Andrea G. Russo^1^, Assunta Ciarlo^2^, Sara Ponticorvo^2,3^, Francesco Di Salle^2,4^, Gioacchino Tedeschi^1^, Fabrizio Esposito^1,*^

^1^ Department of Advanced Medical and Surgical Sciences, School of Medicine and Surgery, University of Campania "Luigi Vanvitelli”, Naples, Italy

^2^ Department of Medicine, Surgery and Dentistry, "Scuola Medica Salernitana", University of Salerno, Baronissi, Salerno, Italy

^3^ Center for Magnetic Resonance Research, Department of Radiology, University of Minnesota, Minneapolis, MN, USA

^4^ Department of Diagnostic Imaging, University Hospital “San Giovanni di Dio e Ruggi D’Aragona”, Salerno, Italy

*** Corresponding author:**

Fabrizio Esposito, Ph.D.

Department of Advanced Medical and Surgical Sciences

University of Campania “Luigi Vanvitelli”

Piazza Luigi Miraglia, 2, Naples 80138, Italy.

Email: fabrizio.esposito@unicampania.it

**Materials and Methods**

*Stimuli and experimental procedure*

Participants listened to a short story (11:50 min) entitled “Storia di Gianna e delle sue chiavi” (Story of Gianna and her keys) written by Carlo Santulli and read by Silvia Cecchini, available at the website of “Progetto Babele Rivista Letteraria” ^[[1]](#footnote-1)^. The audio narrative was spoken at an average rate of 156 words per minute and fitted in the recommended word per minute rate for audiobooks ^1^. Audacity 2.03 ^[[2]](#footnote-2)^ was used to obtain the reversed version by reversing the original audio waveform.

Participants listened to the story, as well as to its reversed version, while in the MRI scanner. Considering the twenty-seven participants, fifteen participants started with the non-reversed stimulus and twelve participants with the reversed speech stimulus. Before entering the scanner, participants were instructed to listen to the stimuli as carefully as possible. A short break separated the two versions of the story. Stimuli were presented using a custom script written in Python 2.7 with the use of the PsychoPy2 module ^2^ via MRI-compatible earphones ^[[3]](#footnote-3)^.

*Image acquisition and functional MRI pre-processing*

MRI acquisition was performed with a 3T scanner (Magnetom Skyra, Siemens Healthcare, Germany) equipped with a 20-channel parallel head coil. Structural data were obtained using a three-dimensional T1w Magnetization Prepared Rapid Gradient-Echo (MPRAGE), whereas a multi-band ^3–5^ repeated gradient-echo echo-planar imaging (EPI) sequence of 750 volumes was used to obtain functional data. Full details on the sequence parameters can be found in Russo et al. ^6^.

MRI data of the twenty-seven healthy ^[[4]](#footnote-4)^ volunteers were pre-processed using FLS ^7^ and the Data Processing Assistant for Resting-State fMRI toolkit ^[[5]](#footnote-5)^ ^8^ that is implemented in MATLAB R2021a ^[[6]](#footnote-6)^ and it is based on SPM12 routines ^[[7]](#footnote-7)^.

First, fMRI data underwent visual inspection and correction of the EPI geometrical distortions with FLS TOPUP routine^9,10^. Then, functional and structural data were provided in input to a DPARSF procedure. Slice timing correction, motion correction, and high-pass filtering (cut-off to 0.008 Hz) were applied to the fMRI data. Both anatomical and functional images were spatially normalized to the standard Montreal Neurological Institute (MNI) template and functional scans were resampled to 2 × 2 × 2 mm voxel sizes. White matter (WM), cerebrospinal fluid (CSF) mean signals, Friston-24 movement parameters ^11^, and the motion-related spikes associated with a framewise displacement (FD) higher than 0.5 ^12^ were modeled and regressed out from fMRI time-series. Finally, functional images were spatially smoothed using an isotropic 4 mm full-width at half-maximum Gaussian kernel.

One subject (subject #18) was excluded from the fMRI data analysis due to excessive motion (translation/rotation higher than 3mm/3 degree) in one of the two fMRI runs, resulting in a group of 26 participants (20 females). The 3mm/3 degree was chosen as threshold as it corresponds to the voxel dimension (rounded up), as suggested in ^11^.

The mean FD of each participant was below 0.5 mm ^12^.

*Estimation of the next-word probability*

Next-word probability and corresponding saliency scores were estimated using a pre-trained autoregressive language model, namely GPT-2 ^13^. In particular, we used a version of GPT-2 for the Italian language, called “GePpeTto” ^14^, that has been trained using the original settings of the GPT-2 architecture on a collection of Italian texts, amounting to almost 13GB, extracted from Wikipedia and ItWac corpus ^15^. GePpeTto model corresponds to the small version of the GPT-2 and it includes 12 layers, 117M parameters, and a vocabulary of 30,000 tokens. Full details about the text pre-processing, the model parameters, and its training and testing can be found in De Mattei et al. ^14^. The model is implemented in the Hugging Face environment ^16^ and custom python scripts were used to extract all the data used for the fMRI data modeling. To be processed by the GePpeTto model, the raw text of the narrative, including all the punctuation marks except for the quotation marks (deemed not interpretable during the listening), was converted into tokens that were words or sub-words. GPT-2 model architecture relies on a subword-level vocabulary, namely the byte pair encoding ^28^, that aims at optimizing the performances. Therefore, the tokenization procedure could return a token that is either a full word (e.g., “cane”, dog) or a sub-word (e.g., “automatiche”: “auto”+“matiche”, automatic: auto+matic).

*Functional MRI data analysis*

In order to model the fMRI data, for each word of the narrative the time onset (timestamp), the word duration (WD), the lexical frequency (LF), and the word sound (WS) were estimated. In particular, the timestamps and durations were calculated using Speechmatics^[[8]](#footnote-8)^, the log-transformed LFs were extracted from a web corpus of the Italian containing about 4.9 billion words, namely the itTenTen16 corpus^[[9]](#footnote-9)^, and the WS values were obtained by estimating the root mean square value of the audio signal between the onset and the offset of the word itself.

Both surprisal values and saliency scores vectors were used as predictors of interest in two separate GLM where the dependent variable was the average neural signal of the 1000 region-of-interest (ROI) of the 7-networks Schaefer's parcellation ^20^ for both speech conditions. An ROI-based analysis was selected (over a voxel-based analysis) to mitigate the computational costs (especially in the case of saliency scores analysis). For both GLM analyses only the time points characterized by the presence of the audio stimulus were retained, thus resulting in a set of functional data of 709 volumes, and the contrast between the FW and the BW conditions was evaluated.

All the statistical analyses were repeated six times by using each time the surprisal values and the saliency scores associated with a specific time window.

*Functional MRI data analysis: Surprisal*

Before entering the mixed-effects GLM analysis, all the predictors were processed to account for the hemodynamic delay and the temporal resolution of the acquisition. In detail, the vector of surprisal values, LF, WD, and WS were provided as input to the “compute_regressor” function from the “nilearn” python package^[[10]](#footnote-10)^ ^21^ so that the score data were convolved with the standard hemodynamic response function (HRF) of SPM ^[[11]](#footnote-11)^. The HRF represents the conventional approach for modeling the neural dynamics in the BOLD signals induced by a stimulus and it is derived from a Gamma distribution. The convolution operation, that is an integral expressing of the amount of overlap of one function “g” as it is shifted over another function “f”, "blends" the raw predictors with the HRF thus allowing to best account for the slower dynamics of the BOLD response. As part of the convolution procedure, the resulting values are also downsampled to the fMRI temporal resolution (1 Hz), yielding a predictor matrix of size 709x4. Due to its variance inflation factor higher than 10, the WD predictor was dropped from the design matrix as suggested in ^22,23^, for which the remaining predictors (i.e., surprisal/saliency, LF, and WS) were used to map the neural signal of both FW and BW conditions. To ensure a punctual correspondence with the audio of the narrative, the surprisal analysis was performed only on the tokens that were also full words.

For each ROI the average fMRI signal was extracted for both speech conditions (i.e., FW and BW), concatenated along time, and used as dependent variable in a mixed-effects GLM analysis performed in MATLAB. As independent variables, the model included one categorical predictor encoding the speech direction (i.e., FW and BW), four fixed-effect predictors (i.e., surprisal, LF, WS, and the interaction between surprisal and speech direction), and participants were considered as random-effect. To assess where the surprisal values significantly explained better the neural signal in the FW condition (i.e., comprehensible speech) compared to the BW condition (i.e., unintelligible speech), for each ROI the statistical value associated with the interaction between the speech direction and the surprisal was stored. The obtained statistical results were considered significant at p<0.05 corrected with the Bonferroni criterion (considering n = 1000 comparisons) and were stored in a volumetric map (where they were assigned to the corresponding atlas’ parcellation) that was eventually projected onto an inflated brain surface in the MNI space for visualization purposes using CAT12 ^[[12]](#footnote-12)^.

*Functional MRI data analysis: Saliency scores*

Saliency scores were analyzed in a two-step mixed-effects GLM. First-level GLM was applied to the average fMRI signal of each ROI and each participant in a sliding window approach separately for the FW and the BW conditions. As saliency scores indicated the importance of the previous words for the next-word prediction, for each time point eligible for this analysis (i.e., preceded by a number of time points equal to or higher than the window length) the preceding fMRI time course contained in the window was encoded in a GLM analysis with a design matrix including all the corresponding saliency scores, LF, and WS values previously convolved with the HRF (see previous paragraph) and downsampled to the fMRI temporal resolution. The regression coefficient associated with the saliency scores was stored, thus resulting in a time series for each atlas’ parcellation and each speech condition. To ensure a punctual correspondence with the narrative, saliency scores vectors associated with sub-words were not considered for the saliency analysis. However, sub-words in the saliency score vector of the full words were not removed but instead were merged to recover the full words. The saliency score assigned to the latter was obtained by averaging the saliency scores of their forming sub-words.

The time series of regression coefficients of both speech conditions were used in a second-level GLM analysis where participants were treated as random-effects and the effect of the speech condition (i.e. FW and BW) was evaluated. The obtained statistical results were considered significant at p<0.05 corrected with the Bonferroni criterion (considering n = 1000 comparisons) and were stored in a volumetric map (where they were assigned to the corresponding atlas parcellation) that was eventually projected onto an inflated brain surface in the MNI space for visualization purposes using CAT12.

| ID | Area | Window size | | | | | | | | | | | | | |
| --- | --- | --- | --- | --- | --- | --- | --- | --- | --- | --- | --- | --- | --- | --- | --- |
|  |  | 15 s | | | 30 s | | 45 s | | 60 s | | 75 s | | | 90 s | |
|  |  | t (36862) | | p | t  (36862) | p | t (36862) | p | t  (36862) | p | t  (36862) | | p | t  (36862) | p |
| 83 | *LH_SomMot_2* | 4.60 | | 4.32E-06 |  |  |  |  |  |  | 4.64 | | 3.49E-06 | 4.42 | 9.85E-06 |
| 84 | *LH_SomMot_3* | 8.82 | | 1.16E-18 | 7.75 | 9.17E-15 | 7.48 | 7.76E-14 | 7.41 | 1.29E-13 | 8.42 | | 3.93E-17 | 7.50 | 6.67E-14 |
| 85 | *LH_SomMot_4* | 7.34 | | 2.16E-13 | 6.57 | 4.99E-11 | 6.17 | 7.10E-10 | 6.05 | 1.49E-09 | 6.99 | | 2.76E-12 | 6.01 | 1.85E-09 |
| 86 | *LH_SomMot_5* | 6.61 | | 3.77E-11 | 5.42 | 5.87E-08 | 5.57 | 2.64E-08 | 4.46 | 8.32E-06 | 5.91 | | 3.39E-09 | 5.64 | 1.70E-08 |
| 87 | *LH_SomMot_6* | 5.32 | | 1.07E-07 | 4.60 | 4.32E-06 | 4.37 | 1.27E-05 | 4.07 | 4.62E-05 | 5.07 | | 3.93E-07 | 4.97 | 6.70E-07 |
| 88 | *LH_SomMot_7* | 6.73 | | 1.74E-11 | 6.28 | 3.39E-10 | 6.11 | 9.77E-10 | 5.25 | 1.51E-07 | 6.16 | | 7.51E-10 | 6.55 | 5.83E-11 |
| 89 | *LH_SomMot_8* | 9.10 | | 9.67E-20 | 8.03 | 9.77E-16 | 7.87 | 3.66E-15 | 7.15 | 8.65E-13 | 8.19 | | 2.68E-16 | 7.97 | 1.63E-15 |
| 90 | *LH_SomMot_9* | 6.31 | | 2.84E-10 | 5.67 | 1.41E-08 | 5.04 | 4.56E-07 | 4.76 | 1.97E-06 | 5.61 | | 2.02E-08 | 6.11 | 1.02E-09 |
| 94 | *LH_SomMot_13* | 7.47 | | 7.90E-14 | 7.41 | 1.31E-13 | 6.47 | 9.79E-11 | 5.88 | 4.22E-09 | 6.58 | | 4.77E-11 | 6.34 | 2.28E-10 |
| 100 | *LH_SomMot_19* | 7.40 | | 1.36E-13 | 7.38 | 1.60E-13 | 5.93 | 3.04E-09 | 5.56 | 2.71E-08 | 5.79 | | 6.96E-09 | 6.31 | 2.74E-10 |
| 101 | *LH_SomMot_20* | 4.28 | | 1.91E-05 | 4.22 | 2.44E-05 |  |  |  |  | 4.15 | | 3.36E-05 | 4.67 | 2.99E-06 |
| 104 | *LH_SomMot_23* |  | |  | 4.23 | 2.36E-05 |  |  |  |  |  | |  | 4.09 | 4.30E-05 |
| 124 | *LH_SomMot_43* | 4.62 | | 3.89E-06 | 4.99 | 6.22E-07 |  |  | 4.28 | 1.86E-05 | 4.63 | | 3.67E-06 | 5.26 | 1.48E-07 |
| 182 | *LH_DorsAttn_Post_10* | 4.76 | | 1.98E-06 | 4.10 | 4.22E-05 |  |  |  |  |  | |  |  |  |
| 184 | *LH_DorsAttn_Post_12* | 4.84 | | 1.32E-06 | 4.09 | 4.36E-05 |  |  |  |  | 4.08 | | 4.57E-05 | 4.38 | 1.19E-05 |
| 185 | *LH_DorsAttn_Post_13* | 4.68 | | 2.87E-06 | 4.16 | 3.23E-05 | 4.22 | 2.50E-05 | 4.31 | 1.66E-05 |  | |  |  |  |
| 223 | *LH_DorsAttn_FEF_1* | 5.06 | | 4.14E-07 | 4.89 | 1.03E-06 |  |  |  |  |  | |  | 5.24 | 1.57E-07 |
| 243 | *LH_SalVentAttn_TempOcc_1* | 7.40 | | 1.43E-13 | 6.81 | 9.63E-12 | 5.62 | 1.88E-08 | 5.12 | 3.11E-07 | 6.03 | | 1.62E-09 | 6.27 | 3.76E-10 |
| 244 | *LH_SalVentAttn_TempOcc_2* | 7.34 | | 2.12E-13 | 7.08 | 1.42E-12 | 5.56 | 2.75E-08 | 5.34 | 9.40E-08 | 5.59 | | 2.28E-08 | 6.11 | 1.03E-09 |
| 250 | *LH_SalVentAttn_FrOperIns_6* | 4.20 | | 2.72E-05 |  |  |  |  |  |  |  | |  |  |  |
| 310 | *LH_Limbic_TempPole_8* | 6.17 | | 7.10E-10 | 5.82 | 6.04E-09 | 4.61 | 3.99E-06 |  |  | 5.28 | | 1.31E-07 | 4.86 | 1.20E-06 |
| 321 | *LH_Cont_Par_4* | 4.17 | | 3.01E-05 | 4.24 | 2.21E-05 |  |  |  |  |  | |  |  |  |
| 336 | *LH_Cont_Temp_4* | 5.18 | | 2.25E-07 | 5.17 | 2.41E-07 | 4.51 | 6.57E-06 | 4.09 | 4.32E-05 | 4.60 | | 4.16E-06 |  |  |
| 342 | *LH_Cont_PFCl_5* |  | |  |  |  |  |  |  |  |  | |  | 4.26 | 2.07E-05 |
| 355 | *LH_Cont_PFCl_18* | 4.14 | | 3.43E-05 |  |  |  |  |  |  |  | |  | 4.19 | 2.76E-05 |
| 375 | *LH_Default_Par_1* | 5.38 | | 7.38E-08 | 4.57 | 4.88E-06 | 4.13 | 3.59E-05 |  |  | 4.29 | | 1.80E-05 | 4.68 | 2.91E-06 |
| 376 | *LH_Default_Par_2* | 5.07 | | 3.93E-07 | 4.53 | 5.94E-06 |  |  |  |  |  | |  | 4.22 | 2.40E-05 |
| 377 | *LH_Default_Par_3* | 5.64 | | 1.72E-08 | 5.26 | 1.45E-07 | 4.24 | 2.29E-05 |  |  |  | |  | 4.75 | 2.01E-06 |
| 378 | *LH_Default_Par_4* | 6.81 | | 9.82E-12 | 6.39 | 1.72E-10 | 6.06 | 1.36E-09 | 5.09 | 3.59E-07 | 5.29 | | 1.25E-07 | 4.62 | 3.79E-06 |
| 379 | *LH_Default_Par_5* | 6.27 | | 3.76E-10 | 5.63 | 1.81E-08 | 4.66 | 3.12E-06 |  |  | 4.20 | | 2.67E-05 |  |  |
| 380 | *LH_Default_Par_6* | 4.45 | | 8.53E-06 |  |  |  |  |  |  |  | |  |  |  |
| 382 | *LH_Default_Par_8* | 6.68 | | 2.35E-11 | 6.49 | 8.90E-11 | 6.11 | 9.82E-10 | 4.97 | 6.61E-07 | 5.10 | | 3.49E-07 |  |  |
| 383 | *LH_Default_Par_9* | 7.50 | | 6.32E-14 | 6.94 | 3.88E-12 | 5.45 | 5.07E-08 |  |  | 5.00 | | 5.63E-07 |  |  |
| 384 | *LH_Default_Par_10* | 5.81 | | 6.13E-09 | 4.83 | 1.37E-06 |  |  |  |  |  | |  |  |  |
| 385 | *LH_Default_Par_11* | 4.26 | | 2.08E-05 |  |  |  |  |  |  |  | |  |  |  |
| 386 | *LH_Default_Par_12* | 6.95 | | 3.83E-12 | 6.18 | 6.39E-10 | 6.56 | 5.38E-11 | 4.66 | 3.14E-06 | 4.83 | | 1.40E-06 |  |  |
| 388 | *LH_Default_Par_14* | 7.08 | | 1.43E-12 | 6.49 | 8.68E-11 | 5.85 | 4.99E-09 |  |  | 5.33 | | 9.68E-08 |  |  |
| 389 | *LH_Default_Par_15* | 5.77 | | 7.92E-09 | 5.13 | 2.90E-07 |  |  |  |  |  | |  |  |  |
| 391 | *LH_Default_Par_17* | 6.30 | | 3.09E-10 | 6.07 | 1.29E-09 | 5.93 | 3.10E-09 |  |  | 4.51 | | 6.60E-06 |  |  |
| 393 | *LH_Default_Par_19* | 5.09 | | 3.66E-07 | 4.42 | 9.94E-06 |  |  |  |  |  | |  |  |  |
| 394 | *LH_Default_Temp_1* | 6.86 | | 6.98E-12 | 6.35 | 2.17E-10 | 4.43 | 9.60E-06 |  |  | 4.30 | | 1.71E-05 | 4.18 | 2.97E-05 |
| 395 | *LH_Default_Temp_2* | 4.77 | | 1.85E-06 | 4.34 | 1.41E-05 |  |  |  |  |  | |  |  |  |
| 397 | *LH_Default_Temp_4* | 6.83 | | 8.78E-12 | 6.81 | 9.73E-12 | 5.16 | 2.50E-07 |  |  | 4.26 | | 2.03E-05 | 4.48 | 7.63E-06 |
| 398 | *LH_Default_Temp_5* | 6.55 | | 5.73E-11 | 6.13 | 8.96E-10 | 4.73 | 2.26E-06 |  |  | 4.88 | | 1.07E-06 | 5.27 | 1.36E-07 |
| 399 | *LH_Default_Temp_6* | 6.71 | | 1.91E-11 | 6.73 | 1.75E-11 | 5.72 | 1.07E-08 | 4.34 | 1.40E-05 | 4.78 | | 1.79E-06 |  |  |
| 400 | *LH_Default_Temp_7* | 7.32 | | 2.53E-13 | 7.01 | 2.48E-12 | 6.08 | 1.21E-09 | 5.03 | 4.80E-07 | 5.77 | | 7.83E-09 | 6.54 | 6.36E-11 |
| 401 | *LH_Default_Temp_8* | 4.57 | | 5.01E-06 | 4.28 | 1.91E-05 |  |  |  |  |  | |  |  |  |
| 402 | *LH_Default_Temp_9* | 9.53 | | 1.72E-21 | 9.70 | 3.31E-22 | 9.14 | 6.65E-20 | 7.53 | 5.33E-14 | 7.82 | | 5.30E-15 | 7.08 | 1.49E-12 |
| 403 | *LH_Default_Temp_10* | 7.71 | | 1.29E-14 | 7.36 | 1.84E-13 | 6.45 | 1.13E-10 | 5.11 | 3.27E-07 | 5.93 | | 3.06E-09 | 5.33 | 9.91E-08 |
| 404 | *LH_Default_Temp_11* | 9.05 | | 1.51E-19 | 8.25 | 1.60E-16 | 7.81 | 6.08E-15 | 7.13 | 1.02E-12 | 7.61 | | 2.89E-14 | 7.64 | 2.23E-14 |
| 405 | *LH_Default_Temp_12* | 5.57 | | 2.61E-08 | 5.02 | 5.08E-07 | 4.43 | 9.31E-06 |  |  | 4.64 | | 3.46E-06 | 4.65 | 3.35E-06 |
| 406 | *LH_Default_Temp_13* | 7.90 | | 2.86E-15 | 7.60 | 2.94E-14 | 6.55 | 5.73E-11 | 5.28 | 1.30E-07 | 6.01 | | 1.85E-09 | 6.25 | 4.19E-10 |
| 407 | *LH_Default_Temp_14* | 8.65 | | 5.42E-18 | 8.05 | 8.40E-16 | 7.22 | 5.46E-13 | 5.56 | 2.68E-08 | 6.62 | | 3.68E-11 | 7.77 | 7.93E-15 |
| 408 | *LH_Default_Temp_15* | 9.26 | | 2.21E-20 | 8.57 | 1.11E-17 | 8.78 | 1.70E-18 | 8.16 | 3.54E-16 | 8.46 | | 2.88E-17 | 7.48 | 7.41E-14 |
| 409 | *LH_Default_Temp_16* | 7.24 | | 4.55E-13 | 7.01 | 2.41E-12 | 6.14 | 8.41E-10 | 5.28 | 1.33E-07 | 6.27 | | 3.76E-10 | 5.57 | 2.50E-08 |
| 410 | *LH_Default_Temp_17* | 6.51 | | 7.78E-11 | 6.40 | 1.54E-10 | 5.31 | 1.11E-07 | 4.45 | 8.44E-06 | 4.86 | | 1.16E-06 | 5.46 | 4.78E-08 |
| 411 | *LH_Default_Temp_18* | 10.47 | | 1.28E-25 | 9.75 | 1.99E-22 | 8.71 | 3.16E-18 | 7.79 | 7.12E-15 | 8.74 | | 2.51E-18 | 8.53 | 1.52E-17 |
| 412 | *LH_Default_Temp_19* | 7.61 | | 2.86E-14 | 7.31 | 2.72E-13 | 6.22 | 5.11E-10 | 5.28 | 1.28E-07 | 5.96 | | 2.50E-09 | 6.09 | 1.12E-09 |
| 413 | *LH_Default_Temp_20* | 8.19 | | 2.69E-16 | 8.20 | 2.50E-16 | 6.83 | 8.72E-12 | 6.18 | 6.40E-10 | 6.90 | | 5.14E-12 | 6.50 | 7.92E-11 |
| 414 | *LH_Default_Temp_21* | 6.74 | | 1.60E-11 | 6.48 | 9.21E-11 | 5.87 | 4.34E-09 | 4.67 | 3.05E-06 | 5.51 | | 3.63E-08 | 5.40 | 6.88E-08 |
| 415 | *LH_Default_Temp_22* | 8.10 | | 5.67E-16 | 8.24 | 1.71E-16 | 6.91 | 5.05E-12 | 6.41 | 1.49E-10 | 6.70 | | 2.11E-11 | 6.84 | 8.12E-12 |
| 422 | *LH_Default_PFC_7* | 4.58 | | 4.65E-06 | 5.18 | 2.28E-07 | 4.79 | 1.65E-06 |  |  | 4.34 | | 1.43E-05 | 4.33 | 1.51E-05 |
| 430 | *LH_Default_PFC_15* |  | |  | 4.12 | 3.78E-05 |  |  |  |  |  | |  | 4.70 | 2.56E-06 |
| 432 | *LH_Default_PFC_17* |  | |  | 4.28 | 1.88E-05 |  |  |  |  |  | |  | 4.44 | 8.94E-06 |
| 436 | *LH_Default_PFC_21* | 4.45 | | 8.54E-06 | 4.26 | 2.08E-05 |  |  |  |  |  | |  | 4.73 | 2.26E-06 |
| 441 | *LH_Default_PFC_26* | 4.18 | | 2.90E-05 |  |  |  |  |  |  | 4.56 | | 5.04E-06 | 4.91 | 8.95E-07 |
| 444 | *LH_Default_PFC_29* | 5.22 | | 1.82E-07 | 5.37 | 7.98E-08 | 4.47 | 7.82E-06 |  |  | 5.65 | | 1.65E-08 | 4.89 | 1.00E-06 |
| 445 | *LH_Default_PFC_30* |  | |  | 4.32 | 1.59E-05 |  |  |  |  | 5.17 | | 2.32E-07 | 4.57 | 4.91E-06 |
| 448 | *LH_Default_PFC_33* | 5.86 | | 4.70E-09 | 5.64 | 1.67E-08 | 4.23 | 2.35E-05 |  |  | 5.02 | | 5.26E-07 | 4.51 | 6.46E-06 |
| 449 | *LH_Default_PFC_34* | 4.93 | | 8.10E-07 | 4.90 | 9.48E-07 |  |  |  |  | 4.34 | | 1.41E-05 |  |  |
| 451 | *LH_Default_PFC_36* | 6.56 | | 5.43E-11 | 5.45 | 5.15E-08 | 4.34 | 1.42E-05 |  |  | 5.32 | | 1.05E-07 | 5.71 | 1.17E-08 |
| 452 | *LH_Default_PFC_37* | 4.89 | | 1.00E-06 |  |  |  |  |  |  |  | |  |  |  |
| 453 | *LH_Default_PFC_38* | 4.52 | | 6.17E-06 |  |  | 4.36 | 1.31E-05 |  |  | 4.22 | | 2.50E-05 |  |  |
| 455 | *LH_Default_PFC_40* | 6.41 | | 1.45E-10 | 5.69 | 1.29E-08 | 4.13 | 3.59E-05 |  |  | 4.97 | | 6.71E-07 | 4.38 | 1.17E-05 |
| 456 | *LH_Default_PFC_41* | 4.53 | | 5.85E-06 |  |  | 4.07 | 4.67E-05 |  |  | 4.32 | | 1.58E-05 |  |  |
| 457 | *LH_Default_PFC_42* | 9.28 | | 1.82E-20 | 8.80 | 1.41E-18 | 6.70 | 2.16E-11 | 5.29 | 1.23E-07 | 6.34 | | 2.30E-10 | 7.55 | 4.32E-14 |
| 458 | *LH_Default_PFC_43* | 7.78 | | 7.45E-15 | 6.74 | 1.59E-11 | 5.59 | 2.35E-08 |  |  | 5.14 | | 2.73E-07 | 4.70 | 2.58E-06 |
| 459 | *LH_Default_PFC_44* | 6.55 | | 5.64E-11 | 5.66 | 1.54E-08 | 5.05 | 4.44E-07 |  |  | 4.94 | | 7.66E-07 | 4.30 | 1.69E-05 |
| 460 | *LH_Default_PFC_45* | 5.57 | | 2.58E-08 | 5.32 | 1.06E-07 | 5.43 | 5.80E-08 |  |  | 5.05 | | 4.42E-07 | 4.27 | 1.93E-05 |
| 462 | *LH_Default_PFC_47* | 4.83 | | 1.40E-06 | 4.14 | 3.50E-05 |  |  |  |  | 4.08 | | 4.59E-05 |  |  |
| 464 | *LH_Default_PFC_49* | 4.68 | | 2.94E-06 |  |  |  |  |  |  |  | |  | 4.69 | 2.69E-06 |
| 470 | *LH_Default_pCunPCC_5* | 4.67 | | 3.08E-06 | 4.75 | 2.09E-06 | 5.24 | 1.60E-07 |  |  | 4.11 | | 3.89E-05 |  |  |
| 473 | *LH_Default_pCunPCC_8* | 4.79 | | 1.64E-06 | 5.08 | 3.72E-07 | 4.82 | 1.41E-06 |  |  | 4.10 | | 4.12E-05 |  |  |
| 475 | *LH_Default_pCunPCC_10* | 4.08 | | 4.59E-05 | 4.27 | 1.93E-05 | 4.84 | 1.29E-06 |  |  |  | |  |  |  |
| 479 | *LH_Default_pCunPCC_14* | 5.74 | | 9.44E-09 | 6.15 | 7.73E-10 | 6.15 | 7.65E-10 | 4.11 | 4.01E-05 | 5.02 | | 5.32E-07 | 4.63 | 3.75E-06 |
| 481 | *LH_Default_pCunPCC_16* | 4.75 | | 2.01E-06 | 4.87 | 1.15E-06 | 4.87 | 1.13E-06 |  |  | 4.63 | | 3.62E-06 | 4.14 | 3.43E-05 |
| 485 | *LH_Default_pCunPCC_20* | 4.31 | | 1.63E-05 | 4.49 | 7.07E-06 | 4.69 | 2.78E-06 |  |  |  | |  | 4.34 | 1.42E-05 |
| 583 | *RH_SomMot_2* | 6.98 | | 3.05E-12 | 6.43 | 1.31E-10 | 6.07 | 1.27E-09 | 5.18 | 2.20E-07 | 6.27 | | 3.72E-10 | 6.68 | 2.40E-11 |
| 585 | *RH_SomMot_4* | 4.38 | | 1.20E-05 | 4.72 | 2.42E-06 | 4.91 | 9.00E-07 | 4.24 | 2.23E-05 | 4.34 | | 1.42E-05 | 4.68 | 2.94E-06 |
| 586 | *RH_SomMot_5* | 6.59 | | 4.58E-11 | 6.26 | 3.79E-10 | 5.62 | 1.97E-08 | 5.19 | 2.16E-07 | 5.97 | | 2.42E-09 | 5.79 | 7.23E-09 |
| 588 | *RH_SomMot_7* | 5.85 | | 5.02E-09 | 5.32 | 1.04E-07 | 5.74 | 9.62E-09 | 5.21 | 1.94E-07 | 5.95 | | 2.77E-09 | 5.89 | 4.01E-09 |
| 590 | *RH_SomMot_9* | 5.79 | | 7.00E-09 | 5.44 | 5.44E-08 | 4.77 | 1.84E-06 |  |  | 5.37 | | 8.08E-08 | 5.18 | 2.20E-07 |
| 591 | *RH_SomMot_10* | 5.06 | | 4.13E-07 | 4.69 | 2.80E-06 | 4.55 | 5.50E-06 |  |  | 4.81 | | 1.49E-06 | 5.47 | 4.52E-08 |
| 593 | *RH_SomMot_12* | 7.44 | | 1.02E-13 | 7.31 | 2.74E-13 | 6.55 | 5.75E-11 | 5.28 | 1.29E-07 | 6.58 | | 4.68E-11 | 6.84 | 8.00E-12 |
| 594 | *RH_SomMot_13* |  | |  | 4.55 | 5.28E-06 | 4.27 | 1.94E-05 |  |  |  | |  | 4.81 | 1.54E-06 |
| 595 | *RH_SomMot_14* | 4.10 | | 4.07E-05 | 4.30 | 1.68E-05 |  |  |  |  | 4.17 | | 3.03E-05 | 4.83 | 1.39E-06 |
| 599 | *RH_SomMot_18* | 4.38 | | 1.18E-05 |  |  |  |  |  |  | 4.25 | | 2.11E-05 | 4.44 | 9.12E-06 |
| 689 | *RH_DorsAttn_Post_5* |  | |  |  |  |  |  |  |  |  | |  | 4.40 | 1.09E-05 |
| 694 | *RH_DorsAttn_Post_10* | 4.68 | | 2.94E-06 | 4.15 | 3.26E-05 | 4.28 | 1.83E-05 |  |  |  | |  | 4.80 | 1.62E-06 |
| 746 | *RH_SalVentAttn_TempOccPar_1* | 5.16 | | 2.47E-07 | 5.54 | 3.03E-08 | 4.84 | 1.28E-06 | 4.29 | 1.82E-05 | 4.83 | | 1.39E-06 | 6.06 | 1.38E-09 |
| 747 | *RH_SalVentAttn_TempOccPar_2* |  | |  | 4.17 | 3.01E-05 |  |  |  |  |  | |  | 5.24 | 1.62E-07 |
| 749 | *RH_SalVentAttn_TempOccPar_4* |  | |  |  |  |  |  |  |  |  | |  | 4.23 | 2.31E-05 |
| 764 | *RH_SalVentAttn_PrC_1* |  | |  |  |  |  |  |  |  |  | |  | 5.07 | 4.02E-07 |
| 835 | *RH_Limbic_TempPole_10* | 5.96 | | 2.52E-09 | 6.19 | 6.01E-10 | 5.36 | 8.54E-08 |  |  | 4.88 | | 1.06E-06 | 5.02 | 5.17E-07 |
| 862 | *RH_Cont_Temp_3* |  | |  |  |  |  |  |  |  |  | |  | 4.12 | 3.82E-05 |
| 863 | *RH_Cont_Temp_4* |  | |  |  |  |  |  |  |  |  | |  | 4.27 | 1.93E-05 |
| 878 | *RH_Cont_PFCl_13* |  | |  |  |  |  |  |  |  |  | |  | 4.18 | 2.95E-05 |
| 886 | *RH_Cont_PFCl_21* |  | |  |  |  |  |  |  |  |  | |  | 4.58 | 4.61E-06 |
| 888 | *RH_Cont_PFCl_23* |  | |  |  |  |  |  |  |  |  | |  | 4.69 | 2.74E-06 |
| 890 | *RH_Cont_PFCl_25* | 6.22 | | 5.17E-10 | 5.77 | 8.05E-09 | 4.32 | 1.54E-05 |  |  | 4.12 | | 3.86E-05 | 6.05 | 1.48E-09 |
| 894 | *RH_Cont_PFCl_29* |  | |  |  |  |  |  |  |  |  | |  | 4.98 | 6.35E-07 |
| 911 | *RH_Cont_PFCmp_4* | 4.85 | | 1.23E-06 | 4.53 | 5.99E-06 |  |  |  |  |  | |  | 4.97 | 6.63E-07 |
| 913 | *RH_Default_Par_1* |  | |  |  |  |  |  |  |  |  | |  | 4.64 | 3.44E-06 |
| 915 | *RH_Default_Par_3* | 4.40 | | 1.08E-05 |  |  | 4.10 | 4.16E-05 |  |  |  | |  |  |  |
| 916 | *RH_Default_Par_4* | 4.43 | | 9.27E-06 |  |  |  |  |  |  |  | |  |  |  |
| 918 | *RH_Default_Par_6* | 4.38 | | 1.20E-05 | 4.30 | 1.71E-05 | 4.33 | 1.53E-05 |  |  |  | |  |  |  |
| 920 | *RH_Default_Par_8* | 4.37 | | 1.26E-05 |  |  |  |  |  |  |  | |  |  |  |
| 923 | *RH_Default_Par_11* | 6.00 | | 1.97E-09 | 5.38 | 7.48E-08 | 4.27 | 1.92E-05 |  |  |  | |  |  |  |
| 924 | *RH_Default_Par_12* | 5.02 | | 5.19E-07 | 4.82 | 1.46E-06 | 4.44 | 8.93E-06 |  |  |  | |  |  |  |
| 926 | *RH_Default_Par_14* | 6.03 | | 1.68E-09 | 5.50 | 3.92E-08 |  |  |  |  |  | |  |  |  |
| 928 | *RH_Default_Par_16* | 5.34 | | 9.42E-08 | 4.97 | 6.65E-07 | 4.18 | 2.97E-05 |  |  |  | |  |  |  |
| 929 | *RH_Default_Temp_1* | 6.47 | | 9.85E-11 | 6.29 | 3.28E-10 | 5.04 | 4.71E-07 | 4.12 | 3.76E-05 | 4.44 | | 9.19E-06 | 5.12 | 3.12E-07 |
| 931 | *RH_Default_Temp_3* | 5.18 | | 2.20E-07 | 5.16 | 2.42E-07 | 4.68 | 2.93E-06 |  |  |  | |  |  |  |
| 932 | *RH_Default_Temp_4* | 5.23 | | 1.67E-07 | 5.34 | 9.33E-08 | 4.48 | 7.48E-06 |  |  |  | |  | 4.14 | 3.43E-05 |
| 933 | *RH_Default_Temp_5* | 6.51 | | 7.60E-11 | 5.97 | 2.35E-09 | 4.89 | 9.89E-07 |  |  | 5.16 | | 2.44E-07 | 6.32 | 2.70E-10 |
| 934 | *RH_Default_Temp_6* | 7.28 | | 3.41E-13 | 7.28 | 3.40E-13 | 7.05 | 1.84E-12 | 5.07 | 4.00E-07 | 5.72 | | 1.07E-08 | 6.35 | 2.19E-10 |
| 936 | *RH_Default_Temp_8* | 5.93 | | 3.14E-09 | 5.48 | 4.29E-08 | 5.45 | 5.06E-08 |  |  | 4.49 | | 7.31E-06 | 4.79 | 1.67E-06 |
| 937 | *RH_Default_Temp_9* |  | |  |  |  |  |  |  |  |  | |  | 4.49 | 7.24E-06 |
| 939 | *RH_Default_Temp_11* |  | |  |  |  |  |  |  |  |  | |  | 4.40 | 1.10E-05 |
| 940 | *RH_Default_Temp_12* | 8.84 | | 9.76E-19 | 8.42 | 3.86E-17 | 7.59 | 3.28E-14 | 5.89 | 3.91E-09 | 7.15 | | 9.09E-13 | 8.45 | 2.93E-17 |
| 941 | *RH_Default_Temp_13* | 4.87 | | 1.14E-06 | 5.31 | 1.09E-07 | 5.28 | 1.33E-07 | 4.40 | 1.06E-05 | 4.57 | | 4.87E-06 | 6.07 | 1.33E-09 |
| 942 | *RH_Default_Temp_14* | 4.87 | | 1.10E-06 | 5.29 | 1.23E-07 | 4.91 | 8.97E-07 |  |  | 4.07 | | 4.71E-05 | 5.26 | 1.45E-07 |
| 943 | *RH_Default_Temp_15* | 7.05 | | 1.76E-12 | 6.88 | 6.09E-12 | 5.91 | 3.55E-09 | 4.31 | 1.63E-05 | 5.84 | | 5.36E-09 | 6.83 | 8.54E-12 |
| 944 | *RH_Default_Temp_16* |  | |  | 4.13 | 3.58E-05 |  |  |  |  |  | |  | 4.84 | 1.32E-06 |
| 946 | *RH_Default_Temp_18* | 4.85 | | 1.22E-06 | 5.28 | 1.30E-07 | 4.49 | 7.26E-06 |  |  |  | |  | 5.35 | 9.07E-08 |
| 954 | *RH_Default_PFCv_8* |  | |  |  |  |  |  |  |  |  | |  | 4.47 | 7.90E-06 |
| 956 | *RH_Default_PFCv_10* |  | |  |  |  |  |  |  |  |  | |  | 4.52 | 6.27E-06 |
| 969 | *RH_Default_PFCdPFCm_13* | 4.70 | | 2.66E-06 | 4.56 | 5.12E-06 | 4.35 | 1.35E-05 |  |  | 4.37 | | 1.24E-05 | 4.50 | 6.75E-06 |
| 974 | *RH_Default_PFCdPFCm_18* | 7.01 | | 2.41E-12 | 6.61 | 3.92E-11 | 6.00 | 2.03E-09 | 4.91 | 8.99E-07 | 5.84 | | 5.34E-09 | 6.42 | 1.35E-10 |
| 986 | *RH_Default_pCunPCC_6* | 4.46 | | 8.26E-06 | 4.71 | 2.46E-06 | 5.02 | 5.07E-07 |  |  |  | |  |  |  |
| 987 | *RH_Default_pCunPCC_7* |  | |  |  |  | 4.10 | 4.13E-05 |  |  |  | |  |  |  |
| 988 | *RH_Default_pCunPCC_8* | 4.60 | | 4.19E-06 | 5.16 | 2.53E-07 | 4.69 | 2.80E-06 |  |  |  | |  |  |  |
| 990 | *RH_Default_pCunPCC_10* | 4.11 | | 4.05E-05 | 4.55 | 5.34E-06 | 4.77 | 1.89E-06 |  |  |  | |  |  |  |
| 996 | *RH_Default_pCunPCC_16* | 4.33 | | 1.47E-05 | 4.48 | 7.57E-06 | 4.74 | 2.11E-06 |  |  |  | |  |  |  |
|  | | | | | | | | | | | | | | | |
|  |  | | 15 s | | 30 s | | 45 s | | 60 s | | | 75 s | | 90 s | |
|  | *Total of significant regions* | | 121 | | 114 | | 94 | | 46 | | | 85 | | 103 | |
|  | | | | | | | | | | | | | | | |
|  | **Frequency of activations across time windows** | | | | | | | | | | | | | | |
|  |  | | 1 window | | 2 windows | | 3 windows | | 4 windows | | | 5 windows | | 6 windows | |
|  | *Number of regions* | | 22 (15.68%) | | 16 (11.11%) | | 23 (15.97%) | | 15 (10.42%) | | | 28 (19.44%) | | 40 (27.78%) | |

**Supplementary table 1** Results of the surprisal analysis. In the first part of the table, for each time window a list of the region-of-interest (ROIs), with the corresponding t-statistic and p-value, that elicited significant activations (p<0.05 corrected with the Bonferroni criterion, considering n = 1000 comparisons) is reported.
In the second part of the table, the total number of significant ROIs for each time window is indicated.
In the third part of the table, the number of ROIs that were observed to be significant in a single time window or multiple time windows. The first column of the table indicates the number assigned to the corresponding brain areas in the Schaefer’s parcellation.

| ID | Area | Window size | | | | | |
| --- | --- | --- | --- | --- | --- | --- | --- |
|  |  | 60 s | | 75 s | | 90 s | |
|  |  | t (33174) | p | t (32394) | p | t (31614) | p |
| 84 | LH_SomMot_3 | 5.95 | 2.79E-09 |  |  |  |  |
| 85 | LH_SomMot_4 | 5.34 | 9.38E-08 |  |  |  |  |
| 88 | LH_SomMot_7 | 4.76 | 1.97E-06 | 4.65 | 3.30E-06 | 4.49 | 7.05E-06 |
| 89 | LH_SomMot_8 | 4.84 | 1.28E-06 |  |  |  |  |
| 90 | LH_SomMot_9 | 4.21 | 2.60E-05 |  |  |  |  |
| 178 | LH_DorsAttn_Post_6 | 4.27 | 1.96E-05 |  |  |  |  |
| 337 | LH_Cont_PFCd_1 | 4.23 | 2.39E-05 |  |  |  |  |
| 404 | LH_Default_Temp_11 | 4.11 | 4.01E-05 |  |  |  |  |
| 408 | LH_Default_Temp_15 | 6.51 | 7.54E-11 | 5.21 | 1.89E-07 |  |  |
| 411 | LH_Default_Temp_18 | 5.13 | 2.87E-07 | 4.95 | 7.53E-07 | 4.87 | 1.14E-06 |
| 414 | LH_Default_Temp_21 | 4.81 | 1.53E-06 | 4.47 | 7.86E-06 | 4.74 | 2.19E-06 |
| 588 | RH_SomMot_7 | 4.29 | 1.76E-05 |  |  |  |  |
| 590 | RH_SomMot_9 | 4.13 | 3.56E-05 |  |  |  |  |
| 593 | RH_SomMot_12 | 4.32 | 1.55E-05 | 4.93 | 8.10E-07 | 4.34 | 1.40E-05 |
| 595 | RH_SomMot_14 |  |  |  |  | 4.28 | 1.87E-05 |
| 773 | RH_SalVentAttn_FrOperIns_8 |  |  |  |  | 4.08 | 4.48E-05 |
| 933 | RH_Default_Temp_5 |  |  |  |  | 4.44 | 8.93E-06 |
|  | | | | | | | |
|  |  | 60 s | | 75 s | | 90 s | |
|  | *Total of significant regions* | 14 | | 5 | | 7 | |
|  | | | | | | | |
|  | **Frequency of activations across time windows** | | | | | | |
|  |  | 1 window | | 2 windows | | 3 windows | |
|  | *Number of regions* | 12 (70.59%) | | 1 (5.88%) | | 4 (23.53%) | |

**Supplementary table 2** Results of the saliency scores analysis. In the first part of the table, for each time window a list of the region-of-interest (ROIs), with the corresponding t-statistic and p-value, that elicited significant activations (p<0.05 corrected with the Bonferroni criterion, considering n = 1000 comparisons) is reported.
In the second part of the table, the total number of significant ROIs for each time window is indicated.
In the third part of the table, the number of ROIs that were observed to be significant in a single time window or multiple time windows. The first column of the table indicates the number assigned to the corresponding brain areas in the Schaefer’s parcellation.

1. <http://www.progettobabele.it> <https://www.progettobabele.it/AUDIOFILES/ascolta.php?ID=841> [↑](#footnote-ref-1)
2. <https://www.audacityteam.org/> [↑](#footnote-ref-2)
3. Serene Sound, Resonance Technology, USA [↑](#footnote-ref-3)
4. Available at <https://osf.io/uz6qd/> [↑](#footnote-ref-4)
5. DPARSF 5.0; <http://www.rfmri.org> [↑](#footnote-ref-5)
6. The MathWorks, Inc., Natick, MA [www.mathworks.com](http://www.mathworks.com) [↑](#footnote-ref-6)
7. Wellcome Department of Imaging Neuroscience, London, UK <http://www.fil.ion.ucl.ac.uk/spm/> [↑](#footnote-ref-7)
8. <https://www.speechmatics.com/> [↑](#footnote-ref-8)
9. <https://www.sketchengine.eu/ittenten-italian-corpus/> [↑](#footnote-ref-9)
10. <https://nilearn.github.io> [↑](#footnote-ref-10)
11. <https://www.fil.ion.ucl.ac.uk/spm/> [↑](#footnote-ref-11)
12. <http://www.neuro.uni-jena.de/cat/index.html> [↑](#footnote-ref-12)
